# Supplementary material for: Resolving the full spectrum of human genome variation using Linked-Reads
Source: Genome Res. 2019 Apr;29(4):635–45. doi: 10.1101/gr.234443.118 (PMC6442396; doi:10.1101/gr.234443.118)
Supplement: Supplemental Material [file supp_gr.234443.118_Supplemental_longranger-2.2.zip › longranger-2.2.tar.bz2!/lr2.2-public/lib/stan/cmdstan/stan/doc/api/html/dir_54e1f17c1ac5ea0adfc355c0b89ee8b2.html]

Stan: src/stan/agrad/fwd/matrix Directory Reference


|  |  |
| --- | --- |
|  | Stan  2.5.0  probability, sampling & optimization |


- Main Page
- Related Pages
- Namespaces
- Classes
- Files

All Classes Namespaces Files Functions Variables Typedefs Enumerations Enumerator Friends Macros Pages

- src
- stan
- agrad
- fwd
- matrix

matrix Directory Reference

|  |  |
| --- | --- |
| Files | |
| file | columns\_dot\_product.hpp [code] |
|  | |
| file | columns\_dot\_self.hpp [code] |
|  | |
| file | crossprod.hpp [code] |
|  | |
| file | determinant.hpp [code] |
|  | |
| file | divide.hpp [code] |
|  | |
| file | dot\_product.hpp [code] |
|  | |
| file | dot\_self.hpp [code] |
|  | |
| file | inverse.hpp [code] |
|  | |
| file | log\_determinant.hpp [code] |
|  | |
| file | log\_softmax.hpp [code] |
|  | |
| file | log\_sum\_exp.hpp [code] |
|  | |
| file | mdivide\_left.hpp [code] |
|  | |
| file | mdivide\_left\_ldlt.hpp [code] |
|  | |
| file | mdivide\_left\_tri\_low.hpp [code] |
|  | |
| file | mdivide\_right.hpp [code] |
|  | |
| file | mdivide\_right\_tri\_low.hpp [code] |
|  | |
| file | multiply.hpp [code] |
|  | |
| file | multiply\_lower\_tri\_self\_transpose.hpp [code] |
|  | |
| file | qr\_Q.hpp [code] |
|  | |
| file | qr\_R.hpp [code] |
|  | |
| file | rows\_dot\_product.hpp [code] |
|  | |
| file | rows\_dot\_self.hpp [code] |
|  | |
| file | softmax.hpp [code] |
|  | |
| file | sort.hpp [code] |
|  | |
| file | sum.hpp [code] |
|  | |
| file | tcrossprod.hpp [code] |
|  | |
| file | to\_fvar.hpp [code] |
|  | |
| file | trace\_gen\_quad\_form.hpp [code] |
|  | |
| file | trace\_quad\_form.hpp [code] |
|  | |
| file | typedefs.hpp [code] |
|  | |


---

[ Stan Home Page ]

*© 2011–2014,
Stan Development Team.*
